# Advancements in the Understanding of Small-Cell Neuroendocrine Cervical Cancer: Where We Stand and What Lies Ahead

**DOI:** 10.3390/jpm14050462

**Published:** 2024-04-27

**Authors:** Yan Wang, Hui Qiu, Rongjie Lin, Weiwei Hong, Jiahao Lu, Huan Ling, Xiaoge Sun, Chunxu Yang

**Affiliations:** 1 Life Sciences Institute, Zhejiang University, Hangzhou 310058, China; 2 Key Laboratory of Integrated Oncology and Intelligent Medicine of Zhejiang Province, Hangzhou First People’s Hospital, Hangzhou 310006, China; 3 Hubei Key Laboratory of Tumor Biological Behaviors, Hubei Cancer Clinical Study Center, Department of Radiation and Medical Oncology, Zhongnan Hospital of Wuhan University, Wuhan 430071, China; 4 Department of Radiotherapy, West China Xiamen Hospital of Sichuan University, Xiamen 361021, China; 5 Department of Medical Oncology, The First Affiliated Hospital of Nanchang University, Nanchang 330006, China; 6 Department of Ultrasound in Gynecology and Obstetrics, Zhongnan Hospital of Wuhan University, Wuhan 430071, China; 7 Department of Radiation Oncology, Affiliated Hospital of Inner Mongolia Medical University, Hohhot 750306, China

**Keywords:** small-cell neuroendocrine carcinoma, uterine cervix, pathogenesis, molecular characteristics, treatment

## Abstract

Small-cell neuroendocrine cervical carcinoma (SCNCC) is a rare yet aggressive gynecological malignancy associated with dismal clinical outcomes. Its rarity has led to a limited number of retrospective studies and an absence of prospective research, posing significant challenges for evidence-based treatment approaches. As a result, most gynecologic oncology centers have limited experience with this tumor, emphasizing the urgent need for a comprehensive review and summary. This article systematically reviews the pathogenesis, immunohistochemical and molecular characteristics, prognostic factors, and clinical management of gynecologic SCNCC. We specifically focused on reviewing the distinct genomic characteristics of SCNCC identified via next-generation sequencing technologies, including loss of heterozygosity (LOH), somatic mutations, structural variations (SVs), and microRNA alterations. The identification of these actionable genomic events offers promise for discovering new molecular targets for drug development and enhancing therapeutic outcomes. Additionally, we delve deeper into key clinical challenges, such as determining the optimal treatment modality between chemoradiation and surgery for International Federation of Gynecology and Obstetrics (FIGO) stage I phase patients within a precision stratification framework, as well as the role of targeted therapy within the homologous recombination (HR) pathway, immune checkpoint inhibitors (ICIs), and prophylactic cranial irradiation (PCI) in the management of SCNCC. Finally, we anticipate the utilization of multiple SCNCC models, including cancer tissue-originated spheroid (CTOS) lines and patient-derived xenografts (PDXs), to decipher driver events and develop individualized therapeutic strategies for clinical application.

## 1. Introduction

Small-cell neuroendocrine carcinoma (SCNEC) is a distinct pathological entity that can occur in both lung and extrapulmonary sites, including the endometrium and uterine cervix [1,2,3,4]. In various reports, the median or mean age at diagnosis for small-cell neuroendocrine cervical carcinoma (SCNCC) ranges from 37 to 50 years [5,6,7,8]. SCNCC is a rare subtype, accounting for less than 5% of all cervical cancers [9]. However, it is an aggressive form of cervical cancer that frequently presents with locally advanced disease and metastasis at diagnosis, as well as high recurrence rates [6,7,10,11,12,13]. Common sites of metastasis include the lung, liver, and bone [14].Its aggressive nature is characterized by high invasiveness and a tendency for lymph–vascular space involvement (LVSI) and lymph node metastasis (LNM), despite having a relatively small depth of invasion (DI) and tumor size [15]. When compared to the lymph nodes of patients with other cell types of cervical cancer, there is a significantly lower incidence of lymphoplasmacytic infiltration but a significant increase in the number of unstimulated regional lymph nodes [16].

The clinical presentation of SCNCC closely resembles conventional pathological types of cervical cancer, primarily exhibiting contact bleeding as a characteristic feature [17]. Nonetheless, the routine cervical smear is relatively insensitive and nonspecific in detecting SCNCC [18]. MRI imaging also reveals nonspecific tumor morphology [19]. Consequently, the diagnosis of SCNCC should primarily depend on light microscopy of hematoxylin and eosin sections, in conjunction with consideration of its distinctive clinical behavior. SCNCC shares architectural patterns with other neuroendocrine tumors, including nesting, trabeculae, peripheral palisading, rosette formation, and a common sheet-like growth pattern [20,21,22]. Numerous mitotic figures and extensive necrosis are common features [22]. SCNCCs are comprised of small cells with hyperchromatic nuclei, scant cytoplasm, indistinct cell borders, and a common nuclear molding pattern [21,22]. The nuclear chromatin is finely granular, and the nucleoli are absent or inconspicuous [22]. The nuclei can range in shape from round to oval or spindled [22]. The diagnosis of SCNCC typically requires the expression of at least two neuroendocrine markers. The traditional neuroendocrine markers, neural cell adhesion molecules (CD56) and synaptophysin (Syn), have been identified as the most sensitive neuroendocrine markers available so far, despite CD56 lacking specificity [23]. Conversely, chromogranin A (CgA) is the most specific neuroendocrine marker, but its sensitivity is limited, with positivity seen in only approximately 50% to 60% of SCNCC cases [24]. Other neuroendocrine markers, including neuron-specific enolase (NSE) [25], insulinoma-associated protein 1 (INSM1) [26], and neurogenic differentiation factor 1 (NEUROD1) [27], have also been introduced in the diagnosis and prognosis research of SCNCC. However, their utility requires further validation.

Due to its rarity, the limited publications available on SCNCC primarily consist of descriptive case series and reports, which have resulted in data of varying quality and occasional controversies. Currently, the majority of gynecologic oncology centers possess limited experience with this tumor. This review provides a detailed analysis of the pathogenesis, immunohistochemical characteristics, prognostic factors, and treatment options for SCNCC, aiming to deepen our understanding of the disease and facilitate the development of innovative and effective therapeutic strategies for patients.

## 2. Unraveling the Complex Pathogenesis of Small-Cell Neuroendocrine Cervical Carcinoma (SCNCC)

### 2.1. The Role of Human Papillomavirus Infection: A Critical Pathogenic Factor

Human papillomavirus (HPV) types 16 and 18 are strongly associated with the development of cervical squamous carcinoma [28,29]. Similarly, across the studies listed in Table 1, it has been observed that SCNCC demonstrates a greater susceptibility to HPV-18 infection, followed by HPV-16.

Among these detection methods, polymerase chain reaction (PCR) is commonly used to detect HPV typing, while in situ hybridization is typically employed to determine HPV distribution. However, PCR analysis may not be very sensitive for detecting all HPV DNA types [30]. In recent years, whole-exome sequencing (WES) has been employed for HPV detection and the analysis of integration sites, revealing potential differences in the pathogenic mechanisms associated with HPV-18 and HPV-16 in SCNCC. HPV-18 exhibited integration sites at the 8q24.21 and 14q13.2 chromosomal regions, primarily leading to increased expression of the proto-oncogenes *MYC* and *PVT1* [31]. This has also been confirmed via RNA sequencing analysis of the original SCNCC tumor and organoid [32]. On the other hand, individual HPV-16 integration events have been identified at 20q11.21 [31], 2q24.1 [33], and 17q12 [33], which are located adjacent to the *NR4A2* and *PGAP3* genes [33]. Previous studies have demonstrated recurrent HPV integrations in *NR4A2*, leading to elevated gene expression levels due to copy-number gains [34,35].

**Table 1:** Detection of HPV type, proportion, and integration in small-cell neuroendocrine cervical carcinoma.

Method	Total Number	HPV18 Positive	HPV16 Positive	HPV18 and HPV16 Co-Infection	Integration Events	Reference
Short PCR fragment (SPF10) primer PCR and INNO-LiPA	22	77.30%	18.20%	4.50%	-	[36]
Quantitative multiplex PCR and the NGS genetic testing panel OncoScreen PlusTM (Burning Rock Dx Ltd., Guangzhou, China)	51	92.16%	43.14%	35.29%	-	[37]
PCR	12	0%	0%	0%	-	[30]
PCR and in situ hybridizations	10	60%	0%	0%	-	[38]
In situ hybridization	18	77.78%	5.56%	0%	-	[39]
In situ hybridization	26	40%	28%	0%	-	[12]
Whole-exome sequencing (WES)	15	40%	13%	0%	8q24.21, 14q13.2, 20q11.21	[31]
RNA sequencing	1	100%	0%	0%	8q24.21	[32]
WES	10	50%	20%	0%	2q24.1,17q12	[33]

In the future, it will be crucial to simultaneously analyze large cohorts of patients using multiple detection and analysis methods to accurately understand the mechanisms of HPV-18 and HPV-16 infection in the development of SCNCC. The ultimate aim is to assess whether anti-HPV therapy could effectively enhance the control of these aggressive neoplasms.

### 2.2. Characteristics of Molecular Pathogenesis

Recent advancements in next-generation sequencing technologies, coupled with advanced bioinformatics tools, have revealed distinct genomic characteristics of SCNCC including loss of heterozygosity (LOH), somatic mutations, structural variations (SVs), and microRNAs alterations (Table 2). These genomic alterations are involved in the PI3K/AKT, MAPK, Wnt, and TP53/BRCA pathways [40,41]. When compared to small-cell lung cancer (SCLC), SCNCC samples demonstrated notably higher frequencies of genomic alterations in *PIK3CA* (24% vs. 5.1%), *MYC* (12.7% vs. 6.3%), *ARID1A* (10.1% vs. 4.2%), and MSI-High (3.1% vs. 0.004%). Conversely, they showed lower frequencies of alterations in *TP53* (12.7% vs. 90.1%) and *RB1* (6.3% vs. 70.9%) [42]. The identification of these actionable genomic events presents an opportunity to gain a deeper understanding of the unique natural history of this disease.

#### 2.2.1. Loss of Heterozygosity

Loss of Heterozygosity (LOH) is a critical event in cancer progression, and the majority of these events have been found to be shared between primary tumors and metastatic samples [49]. To date, the use of polymorphic microsatellite markers has revealed that loss of heterozygosity (LOH) at specific gene/chromosomal regions is a frequent genetic event in SCNCC [21,43,44]. LOH at 3p loci, particularly at 3p14, is a common finding in SCNCC [21,43,44]. However, the occurrence of LOH at *TP53* (17p13) and *RB* (13q14) appears to vary among studies [21,43,44]. Additionally, other regions of LOH such as *THRB* (3p24) [44], *APC-MCC* (5q21-q22) [21], *CDKN2* (9p21) [21], and *INT-2* (11q13) [44] have also been detected in SCNCC. Given the limited number, it is imperative to expand the sample size to conduct a comprehensive characterization of LOH in SCNCC.

#### 2.2.2. Somatic Mutations in the PI3K/AKT Signaling Pathway

Whole-exome sequencing has revealed that the *PIK3CA* p.E545K mutation is the most prevalent oncogenic mutation in neuroendocrine carcinoma of the cervix (NECC), occurring in four tumors (27%) [31]. Additionally, next-generation sequencing, utilizing a 637-gene panel, identified *PIK3CA* mutations in three SCNCC cases (30%), specifically including p.E545K, p.G106A, p.N345T, and p.E545D [38]. Concurrently, a 50-gene panel detected *PIK3CA* mutations in eight SCNCC patients (18%), with mutations such as p.E545K, p.E542K, p.H1047Y, p.R88Q, and p.H1047R [45]. Furthermore, an *AKT1* mutation (p.E17K) was identified in one patient [45]. Mutations in the tumor-suppressor gene *PTEN* were also detected, specifically with p.G106A and p.F241S mutations found in four tumors (40%) [38]. Additionally, *PTEN* mutations including p.V53A and p.H64Y were identified in five SCNCC patients [46].

#### 2.2.3. Somatic Mutations in the MAPK Signaling Pathway

G12D and G12V are the two most prevalent *KRAS* mutations. Next-generation sequencing, utilizing a 637-gene panel, identified *KRAS* (p.G12V) in four SCNCC cases (40%), while a 50-gene panel detected *KRAS* (p.G12D, p.G12V, p.G13D) in six patients (14%) [38,45]. Notably, a patient with a *KRAS* (p.G12D) mutation exhibited a complete radiologic response after receiving three cycles of MEK inhibitor trametinib therapy, suggesting a promising strategy [47]. In addition to *KRAS*, mutations in *Erbb2* (p.R663Q) and *c-Myc* (p.A199T) were detected in four SCNCC tumors (40%) [38]. Furthermore, one SCNCC patient (2%) carrying *NRAS* mutations (p.E137D and p.Q61K) and two patients (5%) harboring *MET* mutations (p.M1247V and p.E168D) were also identified [45].

#### 2.2.4. Somatic Mutations in the TP53/BRCA Signaling Pathway

Next-generation sequencing analysis of 520 cancer-related genes revealed that eleven patients harboring mutations in the *TP53* pathway exhibited significantly poorer prognoses compared to those with wild-type genes (*n =* 38), demonstrating a statistically significant difference in three-year overall survival (OS) (33.5% vs. 59.9%, *p* = 0.031) [50]. Additionally, sequencing based on a 637-gene panel identified four cases harboring somatic mutations in caretaker tumor-suppressor genes: *TP53* (p.C238W, p.E271Q, p.C275Y, p.80fs, and p.P80L), *BRCA1* (p.T367I), and *BRCA2* (p.Q1187fs). [38]. Furthermore, *TP53* mutations (p.C275F, p.C176W, p.R110H, p.S241Y, and p.A355V) were observed in five patients (11%), while *RB1* mutation (p.E137D) were detected in one patient (2%) [45]. Interestingly, *TP53* mutations, specifically p.80fs and p.P80L, resulted in a complete absence of P53 protein expression, aligning with the “null” pattern observed in aberrant or mutation-type P53 expression [38]. The insertion of nucleotide AG in the codon 80 of the *TP53* gene led to a frameshift mutation, prematurely terminating translation and resulting in a truncated P53 protein that was not recognized by the P53 antibody [38]. On the other hand, the *TP53* mutations p.C238W and p.C275Y demonstrated aberrant or mutation-type over-expression of P53. The *TP53* p.E271Q mutation, however, exhibited a normal/wild-type P53 expression pattern in this tumor [38].

#### 2.2.5. Somatic Mutations in the Wnt and Other Prominent Somatic Mutations

Next-generation sequencing, utilizing a panel of 637 genes, identified oncogenic driver mutations in either *BCL6* (p.W375C) or *NCOA3* (p.Q1239_1241del) in four (40%) SCNCC tumors [38]. Additionally, mutations in caretaker tumor suppressors *RB1* (p.S751fs) and *ARID1B* (p.K2043fs) were detected in another four (40%) of these tumors, which frequently harbored activating oncogenic mutations [38]. Furthermore, among the 50 cancer-related genes analyzed, mutations were identified in seven genes. Specifically, three SCNCC cases (7%) exhibited *GNAS* mutations (p.R201S, p.R201C, p.R201H), three (7%) harbored *CTNNB1* mutations (p.G34E, p.S45P, p.T41I), two (5%) carried *SMAD4* mutations (p.E330K, p.N316S), one (2%) showed *SMARCB1* mutations (p.A203T), and one (2%) indicated *FBXW7* mutations (p.R479Q) [45]. *SOX2* was identified as a targetable mutated gene in SCNCC [50]. Co-expression of *SOX2* with high-risk (HR)-HPV RNA, as detected via in situ hybridization (RISH) in SCNCC, may represent a distinct subgroup with significantly poorer prognostic outcomes compared to the expression of each alone [51].

#### 2.2.6. Structural Variations

Structural variations (SVs) have a more profound impact on the cancer genome than any other type of somatic genetic alteration, and they are more specific to individual cancer types compared to other genetic alterations, such as single-nucleotide variants [52]. The four somatic structural variations (SVs) identified in SCNCC include a duplication of the region 15:44,801,470–44,881,820, potentially fusing the genes *CTDSPL2* (NM_016396.3) and *SPG11* (NM_025137.4); homology recombination of *CBL* (NM_005188.4), resulting in a potential reverse fusion of exons 8 and 10; deletion of *MUC17* (NM_00104015.2) exon 3; and deletion of TREH (NM_007180.3) intron 4 [33]. Notably, *CBL* is a proto-oncogene that is known to impact JAK2, EGFR, and PI3K signaling pathways [53]. *CTDSPL2* has been reported as a tumor suppressor involved in restraining tumor growth in pancreatic cancer [54]. *MUC17*, one of the twenty-one mucin genes, also exhibits tumor suppressor properties [55]. These findings suggest that structural variants (SVs) may play a pivotal role in the carcinogenesis of SCNCC.

#### 2.2.7. MicroRNAs in SCNCC

Non-coding microRNAs (miRNAs) are ubiquitous players involved in all cancer hallmarks [56]. A study enrolled 44 patients with SCNCC who underwent a radical hysterectomy. Compared to early-stage SCNCC patients (FIGO IB1), seven miRNAs—including has-let-7c, has-miR-10b, has-miR-100, has-miR-125b, has-miR-143, has-miR-145, and has-miR-199a-5p—were significantly downregulated in advanced-stage SCNCC patients (FIGO IB2-IV). Among these, downregulation of six miRNAs—excluding has-miR-10b—was significantly associated with lymph node metastasis and reduced survival in SCNCC. Kaplan–Meier survival analyses revealed that SCNCC patients with low expression of has-miR-100 (*p* = 0.019) and has-miR-125b (*p* = 0.020) exhibited a significant tendency towards a poorer prognosis [48].

We presumed that miRNAs may play critical roles in the pathogenesis of SCNCC, yet there has been a scarcity of miRNA studies in SCNCC until now. In the future, conducting more studies focusing on the role of miRNAs or other non-coding RNAs in SCNCC will deepen our understanding of its pathogenesis.

In summary, the identical gene can simultaneously harbor a diverse array of somatic alterations, including loss of heterozygosity (LOH), somatic mutations, and structural variations (SVs), all of which collectively affect gene expression and function. The cumulative alterations in genetic and signaling pathways, particularly the PI3K/AKT, MAPK, Wnt, and TP53/BRCA pathways, profoundly influence the biological behaviors of SCNCC. By deeply understanding the role of both coding and non-coding driver mutations in the clonal evolution of SCNCC, we can devise more precise and personalized treatment strategies, maximizing treatment effectiveness while minimizing side effects.

## 3. Immunohistochemical Features

Accurately diagnosing small-cell carcinoma prior to treatment is of utmost importance, yet it remains challenging. In addition to morphology and neuroendocrine markers, the utilization of immunohistochemical analysis encompassing various markers, including transcription factors, as well as specific oncogenes and suppressor genes, can significantly aid in establishing an accurate diagnosis, estimating prognosis, and guiding targeted therapy [57].

### 3.1. Oncogenes and Suppressor Genes

Multiple studies on SCNCC have focused on several prominent “star” molecules, specifically oncogenes such as HER-2/neu and epidermal growth factor receptor (EGFR), as well as tumor-suppressor genes like P16, P53, and retinoblastoma (Rb). A study conducted at the University of Texas M.D. Anderson Cancer Center enrolled eighteen patients with SCNCC and revealed that 38.9% were HER-2/neu-positive and 44.4% were EGFR-positive, respectively [58]. Due to the limited sample size, this study did not assess the prognostic importance of these two oncogenes in SCNCC.

Notably, a ten-year study (2007–2017) conducted by the Catholic University of Sacred Heart found that, when integrated with clinical and instrumental data, the expression of P16 may suggest a cervical origin for neuroendocrine cell tumors [59]. P16 is diffusely expressed in the majority of SCNCC tissue, with a positive rate ranging from 88.9% to 100% [9,36,37,38,60]. The P16-cyclinD-Rb pathway, which regulates G1 phase progression, is crucial for tumor development [61]. Alterations in P16 within SCNCC lead to the loss of Rb protein expression, a phenomenon observed frequently in SCNCC and unrelated to HPV type [62]. Notably, in two separate studies, a complete absence of nuclear Rb staining was observed in sixteen cases (73%) [36] and twenty-three cases (92%) [62] of SCNCC. The remaining two tumors exhibited only weak, focal expression [62].

In addition to Rb, the P53 gene is another prominent tumor suppressor frequently disrupted in cancer cells [63]. Across three independent studies, the P53 protein was undetectable in 50%-100% SCNCC tissues [36,64,65], with seven of these patients succumbing to the disease after a median survival of 20 months [64]. Cadherins are tissue-specific cell adhesion molecules that function as tumor suppressors [65]. In a study, N-cadherin expression was absent in all four examined SCNCC cases, while P-cadherin expression was absent in three tumors (75%) [65]. Conversely, E-cadherin was expressed in three cases (75%) [65]. These findings suggest that N-cadherin may play a crucial role as a tumor-suppressor gene in SCNCC, necessitating further investigation with larger patient cohorts to independently validate this observation.

### 3.2. Immune and Damage Repair Markers

PD-L1 is frequently upregulated in multiple malignancies [66]. In two independent studies, positive PD-L1 expression was observed in twenty-two (51.16%) [67] and seven SCNCC cases (70%) [68], with an average combined positive score of 6.82 [67]. Another study enrolled 89 SCNCC patients, revealing that 68.5% of patients had a combined positive score (CPS) of PD-L1 ≥ 1. A positive tumor proportion score (TPS) and immune cell score (ICS) of PD-L1 were detected in 59.6% and 33.7% of patients, respectively [69]. Notably, the PD-L1 CPS was higher in tumor-infiltrating immune cells (r = 0.387, *p* = 0.001) and positively correlated with programmed cell death-1 (r = 0.443, *p* < 0.001) and forkhead box P3 + regulatory T cell (FOXP3 + Treg) infiltration (r = 0.532, *p* < 0.001), indicating a potential synergistic effect with FOXP3 + Treg and other infiltrating immune cells in supporting an adaptive immune response [69]. Furthermore, PD-L1 CPS positivity (HR = 0.363, *p* = 0.039) and ICS positivity (HR = 0.199, *p* = 0.023) were both independent prognostic factors associated with favorable survival [69]. Additionally, PD-L1 ICS positivity served as an independent indicator of recurrence in SCNCC patients and was associated with improved disease-free survival (HR = 0.124, *p* = 0.001) [69]. These findings suggest that PD-L1 positivity may serve as a favorable prognostic factor in SCNCC [69].

The relationship between PD-L1 and mismatch repair (MMR) expression remains controversial. Although PD-L1 expression has been observed in over 10% of tumor cells in certain tumor subsets associated with the loss of MMR expression [68], another study failed to establish a statistical correlation between PD-L1 and MMR status [69]. Notably, pure high-grade NECC, including small-cell and large-cell carcinoma, exhibit microsatellite stability and predominantly lacks PD-L1 expression [51].

Therefore, it is imperative to precisely evaluate PD-L1 expression and stratify SCNCC patients in large-scale prospective studies to assess their prognosis and eligibility for immune checkpoint inhibitor (ICIs) therapy. This tailored approach offers a promising alternative for the treatment of this aggressive disease.

Poly (ADP-ribose) polymerase–1 (PARP-1) is an abundant enzyme in the cell nucleus that regulates genome repair by binding to DNA damage sites and creating the poly (ADP-ribose) post-translational modification [70]. A total of eleven SCNCC specimens were tested for PARP-1 expression, revealing a positive rate of 91%. Among the positive samples, six (55%) demonstrated high expression, while four (36%) exhibited moderate expression. Given the high prevalence of PARP-1 expression in the majority of tested tumors, the consideration of PARP inhibitors in future clinical trials appears warranted [71].

### 3.3. Other Potential Novel Markers

Though thyroid transcription factor 1 (TTF-1) is of no value in distinction from a pulmonary metastasis, a high percentage of positive immunoreactivity, including diffuse staining, was observed in primary NECC [24]. Furthermore, TTF-1, in conjunction with Syn, may serve as a useful discriminator between extrapulmonary small-cell carcinoma (EPSCC) and poorly differentiated carcinomas (PDCs). Positive staining for TTF-1 was observed in nine cases of EPSCC, while no PDC cases demonstrated positivity (*p* = 0.034). Meanwhile, immunoreactivity for Syn was detected in twenty EPSCC cases and in two cases of PDC with neuroendocrine differentiation (*p* = 0.002). Ultimately, twenty-five (68%) cases were diagnosed as EPSCC, nine cases of which coexisted with non-small-cell carcinoma [72]. Moreover, the expression of somatostatin receptor subtypes (SST2-SST5) suggests a potential role for somatostatin analogues (SSAs) in the diagnosis and treatment of NECC patients [59].

In conclusion, the markers mentioned above enhance our understanding of IHC features, facilitating the diagnosis of SCNCC and providing a deeper insight into prognosis and the potential use of ICIs and targeted therapy such as HER-2, EGFR, and PARP inhibitors. Nonetheless, these findings necessitate further validation through both retrospective and prospective investigations encompassing larger sample sizes.

## 4. Prognostic Factors

SCNCC is considered the most lethal type of cervical cancer due to its aggressive nature and resistance to current treatment regimens [13,73,74]. A survey conducted in the United States involving 188 patients diagnosed with SCNCC revealed that the five-year disease-specific survival rates were 36.8% for FIGO stages I-IIA, 9.8% for stages IIB-IVA, and 0% for stage IVB [75]. In Japan, a study evaluated 65 SCNCC patients between 2003 and 2016, reporting a five-year OS of 49% for all patients and, specifically, 60%, 50%, and 0% for patients with FIGO 2018 stages I-IIA, IIB-IVA, and IVB, respectively [76]. Another analysis enrolling 52 SCNCC patients from 25 medical centers in Japan showed the four-year progression-free survival (PFS) rates were IB1, 59%; IB2, 68%; IIB, 13%; and IIIB, 17%; the four-year OS rates were IB1, 63%; IB2, 67%; IIB, 30%; IIIB, 29%; and IVB, 25% (FIGO 1994) [77]. Similar trends were reported by Zheng et al. in a cohort of 72 Chinese SCNCC patients, treated between 1995 and 2010 at Sun Yat-sen Memorial Hospital, the Cancer Center of Sun Yat-Sen University, and the First Affiliated Hospital of Shantou University Medical College in China. The three-year OS rates were as follows: 100% for stage IA, 62% for stage IB1, 53% for stage IB2, 36% for stage IIA, 29% for stage IIB, 50% for stage IIIB, and 0% for stage IVA [78].

The FIGO staging system has been validated as a prognostic factor that influences both overall and disease-free survival in patients with SCNCC [75,79,80,81,82,83,84,85,86,87,88,89,90,91]. Lymph node metastasis has also been identified as an independent adverse prognostic factor in some reports [81,85,88,90,92], although this finding is not consistent across univariate or multivariate analyses [75,80,87,89,91,93]. The role of age as a prognostic factor remains controversial, as age at diagnosis did not reach significance in a univariate analysis [80]. Furthermore, varying cut-off values for age as an adverse factor have been proposed, such as ≥45, ≥49 and ≥65 years [82,92,94]. However, a study enrolling 130 histologically confirmed SCNCCs found that decreased survival in those with early-stage disease was associated with being older than 60 years at diagnosis (hazard ratio (HR) 4.9; *p* = 0.007). Among patients with advanced-stage disease, decreased survival was associated with being both older than 60 years and younger than 45 years at diagnosis (older than 60 years: HR, 9.9; *p* < 0.001 and younger than 45 years: HR, 3.4; *p* = 0.035) [95].

In addition, a retrospective study involving 93 patients found that tumor size, the depth of stromal invasion, and the treatment modality, including adjuvant radiotherapy, did not significantly impact OS in either univariate or multivariate analyses [80,94]. Nevertheless, other studies have identified specific clinicopathological characteristics that can predict an adverse prognosis for SCNCC patients, including a tumor diameter of >4 cm, deep stromal invasion (>2/3), positive parametrial invasion, positive resection margins, and positive CgA [83,84]. Furthermore, smoking has been reported as a poor prognostic factor for survival among SCNCC patients [89].

In conclusion, the FIGO staging system is a definitive prognostic factor. Although there is controversy, factors including age at diagnosis, lymph node metastasis, tumor diameter, deep stromal invasion, positive parametrial invasion, positive resection margins, CgA positivity, smoking status, and treatment modality can all contribute to predicting the prognosis of SCNCC patients more precisely through stratification in larger cohorts.

## 5. Clinical Treatment

The rarity of SCNCC poses a significant challenge in treating these patients, as there is a dearth of randomized clinical trial information to guide the selection of optimal therapies [96]. Consequently, the majority of available data for this disease primarily originates from retrospective series conducted by large institutions.

### 5.1. Which Is the Preferred Treatment for Early-Stage FIGO I Phase: Chemoradiation or Surgery?

For patients with FIGO 2018 stage IB3 SCNCC (tumor size > 4 cm), it is recommended to undergo concomitant cisplatin-based chemoradiation (CCRT), followed by brachytherapy [24,97]. As for patients with FIGO 2018 stage IA-IB2 SCNCC (tumor size ≤ 4 cm), the principal treatment involves a radical hysterectomy (RH) combined with pelvic lymph node dissection (PLND) [24,97,98]. The open surgical approach, rather than minimally invasive surgery (MIS), should be considered the gold standard for the surgical management of early cervical cancer [98]. Nevertheless, the significance of surgery in the treatment of early-stage SCNCC has been questioned in certain research studies.

A noteworthy study reported that, among patients with stages IA-IIB (*n* = 146), those who underwent surgical treatment (encompassing primary surgery with or without adjuvant therapy, neoadjuvant chemotherapy plus RH-PLND, or perioperative chemotherapy plus RH-PLND) exhibited a tendency towards inferior failure-free survival (FFS) (41.2% versus 60.5%, *p* = 0.086) and cancer-specific survival (CSS) (47.9% versus 61.9%, *p* = 0.122) compared to those who did not undergo surgery [81]. Given that surgery is not recommended for patients with FIGO II [24], we speculate that the lack of stratified analysis may have contributed to this trend. Additionally, the majority of views suggest that surgery and chemotherapy are preferred for SCNCC patients with a tumor ≤2 cm and no LVSI [57]. Therefore, further investigation is required to precisely stratify patients to determine whether surgical treatment for FIGO I, particularly for IB2 patients, is inferior to non-surgical treatment.

Furthermore, a retrospective cohort study conducted by the Taiwanese Gynecologic Oncology Group (TGOG) from 1987 to 2009 demonstrated that concurrent radiation therapy and platinum-based chemotherapy with at least five cycles (EP5+) improved FFS and OS compared to primary surgery (*p* = 0.046 for both) [57]. Given that chemoradiation, rather than surgery, has been recommended as the primary strategy for SCNCC patients in the IB3-II phase [24], further confirmation is necessary in larger, preferably prospective, cohorts to stratify patients into IA–IB1 and IB2 stages and evaluate the superiority of concurrent chemoradiation combined with brachytherapy over primary surgery in each respective stage.

### 5.2. Chemotherapy as the Cornerstone of SCNCC Treatment

Similar to SCLC, chemotherapy is crucial for SCNCC treatment. A retrospective analysis of eleven early-stage SCNCC patients (FIGO stage IA2-IB2) showed significantly higher three-year recurrence-free survival (RFS) and distant recurrence-free survival rates for those treated with initial chemotherapy compared to those without (*p* = 0.045 and *p* = 0.025, respectively) [5]. Cohen et al. found a significant association between the use of chemotherapy (primary, adjuvant, or concurrent with radiation) and improved three-year OS rates in stage IIB-IVA SCNCC patients compared to those without chemotherapy (17.8% vs. 12%, *p* = 0.043, *n* = 37) [75]. Another study showed platinum-based neoadjuvant chemotherapy (NACT) to be effective in preventing distant recurrence. Patients receiving platinum-based NACT combined with radical surgery had significantly lower distant recurrence rates than those receiving radical surgery alone (0/8 vs. 5/9; *p* = 0.029) [79].

Currently, cisplatin (or carboplatin if cisplatin is not tolerated) combined with etoposide (EP) is the recommended chemotherapy strategy for SCNCC in all stages [24,99,100,101]. At least five cycles of EP (EP5+) are strongly recommended as both adjuvant chemotherapy post-surgery and the primary treatment in stages IIB–IVB due to significantly improved five-year RFS, FFS, and CSS compared to other treatments (67.6% vs. 20.9%, *p* < 0.001; 42.9% vs. 11.8%, *p* = 0.041; 45.6% vs. 17.1%, *p* = 0.035) [101]. For concurrent chemoradiation, the initial two cycles of chemotherapy can be administered concurrently with radiation therapy (RT) on days 1 and 22, followed by the subsequent two cycles post-RT [24]. In addition to EP, irinotecan/cisplatin (IP) may also be recommended as a first-line chemotherapy option for locally advanced SCNCC. The five-year OS rates for patients treated with SCNCC (either EP or IP) versus non-SCNCC regimens were 59% and 13%, respectively (*p* < 0.01). No significant difference was observed between IP and EP [76]. However, these findings require further validation in future nationwide, prospective clinical studies.

### 5.3. Targeted Agents and Immune Checkpoint Inhibitors

As previously mentioned, certain genetic alterations have been identified in SCNCC. Targeted therapeutic clinical trials focusing on these alterations and other key targets in SCNEC are summarized in Table 3. The antineoplastic agent ziv-aflibercept, which targets vascular endothelial growth factors (VEGF-A, B, and PlGF), has demonstrated improved PFS in patients with extensive-stage small-cell lung cancer (SCLC) (ClinicalTrials.gov NCT00828139) [102]. Similarly, in recurrent/metastatic SCNCC, anti-angiogenic regimens such as anlotinib, apatinib, and bevacizumab significantly prolonged PFS in first-line treatment compared to controls, with a median PFS of 8 months (range: 2–20 months) versus 3 months (range: 1–10 months), respectively (*p* = 0.025). This trend was also evident among patients who initiated anti-angiogenic treatment after second-line therapy [103]. Consistent with previous studies, Frumovitz et al. found that the combination of a selective topotecan, paclitaxel, and bevacizumab (TPB) for recurrent SCNCC significantly improved PFS over non-TPB regimens, with a median PFS of 7.8 months for TPB and 4 months for non-TPB regimens (hazard ratio HR 0.21, 95% CI 0.09–0.54, *p* = 0.001) [104,105]. Additionally, there were trends towards improved OS [104,105]. Therefore, in the National Comprehensive Cancer Network guidelines Version 1.2023 [24], bevacizumab or an FDA-approved biosimilar is recommended as a first-line, second-line, or subsequent therapy for recurrent or metastatic SCNCC.

In addition to vascular endothelial growth factors, several critical targets within the homologous recombination (HR) pathway, including PARP, ATR, Chk1, and Wee1, have emerged as promising therapeutic candidates for the treatment of small-cell carcinoma [107,108,109,110,111]. A combination of the PARP inhibitor veliparib with temozolomide (TMZ) significantly improved the overall response rate (ORR) in SCLC patients compared to TMZ alone (39% vs. 14%; *p* = 0.016). SLFN11, a biomarker of PARP-inhibitor sensitivity, was found to be associated with prolonged PFS (5.7 vs. 3.6 months; *p* = 0.009) and OS (12.2 vs. 7.5 months; *p* = 0.014) in patients with SLFN11-positive tumors treated with TMZ/veliparib (ClinicalTrials.gov NCT01638546) [107]. In a separate study (ClinicalTrials.gov NCT01286987), among twenty-three evaluated SCLC patients, two achieved a partial response (objective response rate ORR, 9%) with durations of 12.0 and 15.3 weeks, respectively, while four patients exhibited stable disease (SD) lasting at least 16 weeks (clinical benefit rate CBR, 26% ≥16 weeks) [108]. Despite the absence of reported response evaluations of PARP inhibitors for SCNCC, all ten detected SCNCC cases exhibited PARP expression, indicating a potentially promising role for PARP inhibitors in the treatment of SCNCC [117]. An ongoing phase II trial (ClinicalTrials.gov ID NCT04701307) examining the combination of PARP inhibitor niraparib with dostarlimab (anti-PD1) for recurrent SCLC and other high-grade neuroendocrine carcinomas (NEC), including high-grade NECC, may provide further insights into this question. In cells infected with high-risk HPV, phosphorylated-ATR, Chk1/2, and BRCA1 in HR pathways have been observed to be upregulated [118,119]. Collectively, we propose that targeting the HR pathway could potentially be an effective strategy for the treatment of SCNCC. In the future, these HR inhibitors should be employed and verified in SCNCC cases.

The combination of EP with or without atezolizumab (anti-PD-L1) has emerged as a first-line therapy for recurrent/metastatic SCNCC [24]. Immune checkpoint inhibitors (ICIs) combined with chemoradiation show promise in improving clinical outcomes for SCNCC patients. Notably, a stage IVB SCNCC patient with bone and liver metastases achieved prolonged survival through concurrent pembrolizumab (anti-PD1) and chemoradiotherapy [120]. Similarly, a PD-L1-negative patient responded favorably to nivolumab (anti-PD1) and radiotherapy [23]. A reported case of SCNCC responded completely to a combination of nivolumab and ipilimumab (anti-CTLA-4) after a second recurrence, indicating its potential as a new treatment option for recurrent disease [121]. However, recent studies on ICIs monotherapy have shown minimal antitumor activity for SCNCC. In a recent phase II trial of pembrolizumab in rare tumors, there were no responses in seven women with gynecologic extrapulmonary small-cell carcinoma (six cervical, one vulvar) [122]. Given that most patients with high-grade NECC are PD-L1 negative [71], monotherapy may not be sufficient for controlling SCNCC after first-line treatment failure. The combination of anti-PD-1 therapy with ionizing radiation and DNA damage-inducing chemotherapeutics may enhance PD-L1 expression, providing an opportunity for immunotherapy in SCNCC [123].

Taken together, targeting vascular endothelial growth factors and immune checkpoints have been recommended for the treatment of recurrent/metastatic SCNCC. Inhibition of the homologous recombination (HR) pathway is another promising strategy for SCNCC patients. It is conceivable that the combination of these therapeutic approaches may be more effective than monotherapy. Future studies should validate combination strategies utilizing these inhibitors.

### 5.4. Prophylactic Cranial Irradiation (PCI) for SCNCC?

Brain metastases (BrM) are uncommon in EPSCC compared to SCLC. Therefore, prophylactic cranial irradiation (PCI) is probably not indicated for EPSCC [124]. However, SCNCC patients have a higher risk of developing BrM. A retrospective study showed a total of three SCNCC patients (20%) developed BrM [125]. Notably, two of the eight patients with stage I or stage II developed recurrence in the brain, despite having no evidence of extra pelvic disease on initial evaluation [125]. This raises the question of whether SCNCC has a similarly high incidence of central nervous system metastases to SCLC and whether PCI should routinely be recommended for SCNCC treatment. The use of liquid-biopsy-based biomarkers, in combination with current imaging techniques, could enhance the detection of occult BrM [126]. Given that cerebrospinal fluid (CSF) contains higher concentrations of circulating tumor cells (CTC) and circulating tumor DNA (ctDNA) derived from central nervous system tumors than plasma [127], regularly monitoring SCNCC BrM through CTC or ctDNA detection appears promising. If these detections suggest early metastasis, it is extremely likely that the PCI will be strongly recommended in the clinical practice of SCNCC.

## 6. Conclusions and Perspectives

Due to its rarity, the currently available data are primarily descriptive and limited to small groups of case series and reports, resulting in limited-quality data and even controversies. The absence of clinical trials and evidence-based treatment guidelines poses therapeutic challenges for this rare tumor.

Owing to the scarcity of precision stage stratification studies, we are yet to determine the optimal management for the majority of SCNCC patients in early stages, particularly those in IB2, apart from the best prognostic group (<2 cm, no LVSI/deep stromal invasion), who benefit most from RH-PLND. EP is the most recommended chemotherapy strategy for SCNCC across all stages; however, its effectiveness remains limited, and alternative operational chemotherapy regimens are rare.

The absence of established disease models has hindered comprehensive genomic characterization studies and the development of targeted therapies for SCNCC. However, promising platforms such as cancer tissue-originated spheroid (CTOS) lines [128,129] and patient-derived xenografts (PDXs) [130,131] may offer insights into molecular aberrations and effective individualized therapies, including targeted and immune checkpoint inhibitor (ICI) treatments. Notably, BrM is a frequent occurrence in SCNCC, distinguishing it from other EPSCC. Regular monitoring of CTC and ctDNA in CSF can facilitate early screening and diagnosis of BrM, enabling timely PCI.

**Figure 1:**
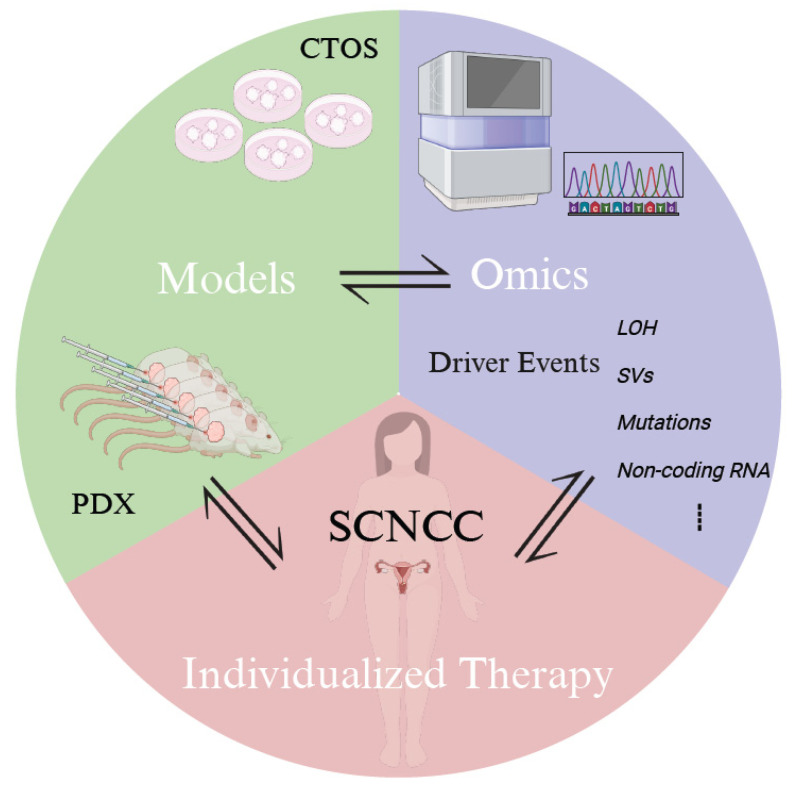
Prospective Individualized Therapeutics in SCNCC. The utilization of high-throughput, integrative “omics” approaches, supported by advanced bioinformatics tools across multiple SCNCC models including CTOS and PDX to decipher driver events and develop individualized therapeutic strategies for clinical application. Abbreviations: CTOS, cancer tissue-originated spheroid; PDX, patient-derived xenograft; SCNCC, small-cell neuroendocrine cervical carcinoma; LOH, loss of heterozygosity; SVs, structural variations.

In summary, these necessities demand larger, precision-stratified, multicenter randomized study designs for SCNCC. Furthermore, the utilization of high-throughput, integrative “omics” approaches, supported by advanced bioinformatics tools across multiple SCNCC models, is imperative for deciphering driver events and developing individualized therapeutic strategies for clinical application (Figure 1).

## Figures and Tables

**Table 2:** Comprehensive overview of genomic alterations detected in small-cell neuroendocrine cervical carcinoma.

Types	PI3K/AKT Signaling Pathway	MAPK Signaling Pathway	TP53/BRCA Signaling Pathway	Wnt Signaling Pathway	Other Signaling Pathways
LOH	3p14–p21 [21,43,44], *INT-2* (11q13) [44]	3p14–p21 [21,43,44], *THRB* (3p24) [44], *INT-2* (11q13) [44]	3p14–p21 [21,43,44], *TP53* (17p13) [43,44], *CDKN2* (9p21) [21],	3p14–p21 [21,43,44], *APC-MCC* (5q21–q22) [21]	*RB* (13q14) [21,43,44]
Somatic Mutations	*PIK3CA* (p.E545K) [31,38,45], *PIK3CA* (p.G106A, p.N345T, p.E545D) [38], *PIK3CA* (p.E542K, p.H1047Y, p.R88Q, p.H1047R) [45], *AKT1* (p.E17K) [45], *PTEN* (p.G106A, p.F241S)[38], *PTEN* (p.V53A, p.H64Y) [46]	*KRAS* (p.G12V) [38,45], *KRAS* (p.G12D) [45,47], *KRAS* (p.G13D) [45], *Erbb2* (p.R663Q) [38], *c-Myc* (p.A199T) [38], *NRAS* (p.E137D, p.Q61K) [45], *MET* (p.M1247V, p.E168D) [45]	*TP53* (p.C238W, p.E271Q, p.C275Y, p.80fs, p.P80L) [38], *TP53* (p.C275F, p.C176W, p.R110H, p.S241Y, p.A355V) [45], *BRCA1* (p.T367I) [38], *BRCA2* (p.Q1187fs) [38], *RB1* (p.E137D) [45]	*CTNNB1* (p.G34E, p.S45P, p.T41I) [45]	*NCOA3*(p.Q1239_1241del) [38], *RB1* (p.S751fs) [38], *NOTCH1* (p.Q2315*nonsense) [38], *BCL6* (p.W375C) [38], *ARID1B* (p.K2043fs) [38], *GNAS* (p.R201S, p.R201C, p.R201H) [45], *SMAD4* (p.E330K, p.N316S) [45], *SMARCB1* (p.A203T) [45], *FBXW7* (p.R479Q) [45],
*Structural Variations*	Homology recombination of *CBL* (NM_005188.4) [33],				Fusing the genes *CTDSPL2* (NM_016396.3) and *SPG11* (NM_025137.4) [33], Deletion of TREH (NM_007180.3) intron 4 [33], Deletion of *MUC17* (NM_00104015.2) exon 3 [33]
*microRNAs*	has-let-7c [48], has-miR-125b [48]	has-miR-199a-5p [48]	has-miR-100 [48], has-miR-143 [48], has-miR-145 [48]	has-miR-10b [48]	

* represents “a mutated site”, indicating a nonsense mutation at the amino acid position Q2315, which means that the codon for the amino acid Q has been altered to become a premature stop codon.

**Table 3:** Clinical trials of targeted therapies and immune checkpoint inhibitors for small-cell neuroendocrine carcinoma.

Target	Agent	Condition or Disease	Phase	Recruitment Status	ClinicalTrials.gov Identifier	Efficacy
PI3K	BKM120	Extensive Stage SCLC	Phase 1	Completed	NCT02194049	-
PI3K and mTOR	PF-05212384	Advanced SCLC	Phase 1	Completed	NCT02069158	
Akt1/2/3	MK-2206	Advanced SCLC	Phase 2	Active, not recruiting	NCT01306045	2 cases PD (100%) [106]
MEK	AZD6244 (Selumetinib)	Advanced SCLC	Phase 2	Active, not recruiting	NCT01306045	1 case PD (100%) [106]
ERBB2	Lapatinib	Advanced SCLC	Phase 2	Active, not recruiting	NCT01306045	1 case SD (100%) [106]
EGFR/ERBB	Afatinib	Stage IV SCLC	Phase 2	Withdrawn (medical decision)	NCT02876081	
MET	ARQ197	Extended SCLC	Phase 2	Terminated (Safety results)	NCT02608411	
Plk1	BI 2536	Sensitive- Relapse SCLC	Phase 2	Completed	NCT00412880	No PR or CR
BET	PLX2853	Advanced SCLC	Phase 1	Completed	NCT03297424	
PARP1/2	Veliparib	Relapsed or Refractory SCLC	Phase 2	Completed	NCT01638546	Improved ORR [107]
PARP1/2	Talazoparib	Advanced or Recurrent SCLC	Phase 1	Completed	NCT01286987	2 cases PR (9%) and 4 cases SD (18%) [108]
PARP1/2	Veliparib	Persistent or Recurrent Carcinoma of the Cervix	Phase 2	Completed	NCT01266447	
PARP1/2	niraparib	SCLC and other High-Grade Neuroendocrine Carcinomas (NEC)	Phase 2	Active, not recruiting	NCT04701307	
PARP1/2	Olaparib	Newly Diagnosed Treatment-Naïve Limited-Stage SCLC	Phase 3	Recruiting	NCT04624204	
PARP1/2	Olaparib	Relapsed SCLC Harboring HR Pathway Gene Mutations	Phase 2	Completed	NCT03009682	
ATR	Berzosertib	Relapsed Platinum-Resistant SCLC	Phase 2	Completed	NCT04768296	
ATR	SC0245	Relapsed Extensive-stage SCLC	Phase 1 and Phase 2	recruiting	NCT05731518	
ATR	M6620	SCLC (*n* = 5) and SCNCC (*n =* 1)	Phase 1 and Phase 2	Active, not recruiting	Olaparib	Tolerable and active; one SCNCC case PD (100%) [109]
ATR	AZD6738	Relapsed SCLC	Phase 2	Completed	NCT03428607	
Chk1	SRA737	Advanced SCLC	Phase 1 and Phase 2	Completed	NCT02797977	11.1% cases PR [110]
Wee1	AZD1775	Advanced SCLC	Phase 1	Completed	NCT02482311	Tolerated, with 33.3% disease control rate (DCR) [111]
Wee1	AZD1775	Relapsed SCLC	Phase 2	Completed	NCT02593019	
Aurora A	MLN8237	Metastatic Castrate Resistant and Neuroendocrine Prostate Cancer	Phase 2	Completed	NCT01799278	Exceptional responders were with tumors suggestive of N-myc and Aurora-A overactivity [112].
Aurora A	JAB-2485	Advanced SCLC	Phase 1 and Phase 2	Recruiting	NCT05490472	
Aurora A	Alisertib	Extensive-stage SCLC	Phase 2	Recruiting	NCT06095505	
Aurora B	AZD2811	Relapsed SCLC	Phase 2	Terminated	NCT03366675	
CDK4/6	Trilaciclib	Extensive-stage SCLC	Phase 1 and Phase 2	Completed	NCT02499770	
CDK7	SY-5609	Advanced SCLC	Phase 1	Completed	NCT04247126	
VEGFR2, VEGFR1, VEGFR3, PDGFRa and c-Kit	Chiauranib	Relapsed or Refractory SCLC	Phase 1	Completed	NCT03216343	
VEGF-A, B and PlGF	ziv-aflibercept	Extensive-stage SCLC	Phase 2	Completed	NCT00828139	Improved PFS [102]
Abl, Src and c-Kit	Dasatinib	Chemo-Sensitive Relapsed SCLC	Phase 2	Completed	NCT00470054	28 cases PD (65.12%)
Wilms Tumor1(WT1)	Galinpepimut-S	Advanced SCLC	Phase 1 and Phase 2	Unknown	NCT03761914	
TP53	Ad.p53-DC vaccines	Extensive-stage SCLC	Phase 2	Completed	NCT00617409	Safe but failed to improve ORRs [113]
TP53	Autologous dendritic cell-adenovirus p53 vaccine	Extensive-stage SCLC	Phase 1 and Phase 2	Completed	NCT00049218	
LSD1	GSK2879552	Relapsed/Refractory SCLC	Phase 1	Terminated	NCT02034123	Poor disease control and an adverse events (AEs) rate [114]
LSD1	Bomedemstat	Extensive-stage SCLC	Phase 1 and Phase 2	Recruiting	NCT05191797	
GSPT1	MRT-2359	SCLC and High Grade NEC	Phase 1 and Phase 2	Recruiting	NCT05546268	
CD3 and DLL3	ZG006	SCLC and NEC	Phase 1 and Phase 2	Recruiting	NCT05978284	
DLL3	Rovalpituzumab tesirine	Advanced or Metastatic SCLC	Phase 3	Completed	NCT03061812	
DLL3	hu3S193	SCLC	Phase 1	Completed	NCT00084799	5 cases PD (100%)
PD-L1 and TGFβ 1	M7824	Relapsed SCLC	Phase 1 and Phase 2	Recruiting	NCT03554473	
PD-1	Pembrolizumab	Small-Cell Ovarian Carcinoma	Phase 2	Recruiting	NCT04602377	
PD-1	Pembrolizumab	Extensive-stage SCLC	Phase 3	Completed	NCT03066778	Improved PFS [115]
PD-1	Pembrolizumab	Extensive-stage SCLC	Phase 1 and Phase 2	Terminated (PI no longer at site)	NCT02331251	
PD-1	Pembrolizumab	Newly Diagnosed Treatment-Naïve Limited-Stage SCLC	Phase 3	Recruiting	NCT04624204	
PD-1 and LAG-3	MGD013 (Tebotelimab)	Extensive-stage SCLC	Phase 1	Completed	NCT03219268	Safe and active (600 mg IV Q2W) [116]
PD1	Dostarlima	SCLC and other High-Grade NEC	Phase 2	Active, not recruiting	NCT04701307	
PD-L1 and CD274	SHR-1316 (Adebrelimab)	Limited-Stage SCLC	Phase 3	Not yet recruiting	NCT05496166	
BET Bromodomain and PD-1	ZEN-3694 Pembrolizumab	Metastatic Prostate Small-Cell Carcinoma	Phase 2	Recruiting	NCT04471974	

## Data Availability

No new data were created or analyzed in this study. Data sharing is not applicable to this article.

## References

[1] Lo Re G., Canzonieri V., Veronesi A., Dal Bo V., Barzan L., Zancanaro C., Trovò M. (1994). Extrapulmonary small cell carcinoma: A single-institution experience and review of the literature. Ann. Oncol..

[2] Cimino-Mathews A., Sharma R., Illei P.B. (2012). Detection of human papillomavirus in small cell carcinomas of the anus and rectum. Am. J. Surg. Pathol..

[3] Randall M.E., Kim J.A., Mills S.E., Hahn S.S., Constable W.C. (1986). Uncommon variants of cervical carcinoma treated with radical irradiation. A clinicopathologic study of 66 cases. Cancer.

[4] Huntsman D.G., Clement P.B., Gilks C.B., Scully R.E. (1994). Small-cell carcinoma of the endometrium. A clinicopathological study of sixteen cases. Am. J. Surg. Pathol..

[5] Zivanovic O., Leitao M.M., Park K.J., Zhao H., Diaz J.P., Konner J., Alektiar K., Chi D.S., Abu-Rustum N.R., Aghajanian C. (2009). Small cell neuroendocrine carcinoma of the cervix: Analysis of outcome, recurrence pattern and the impact of platinum-based combination chemotherapy. Gynecol. Oncol..

[6] Chen J., Macdonald O.K., Gaffney D.K. (2008). Incidence, mortality, and prognostic factors of small cell carcinoma of the cervix. Obstet. Gynecol..

[7] McCusker M.E., Coté T.R., Clegg L.X., Tavassoli F.J. (2003). Endocrine tumors of the uterine cervix: Incidence, demographics, and survival with comparison to squamous cell carcinoma. Gynecol. Oncol..

[8] Dongol S., Tai Y., Shao Y., Jiang J., Kong B. (2014). A retrospective clinicopathological analysis of small-cell carcinoma of the uterine cervix. Mol. Clin. Oncol..

[9] Lu J., Li Y., Wang J. (2022). Small Cell (Neuroendocrine) Carcinoma of the Cervix: An Analysis for 19 Cases and Literature Review. Front. Cell Infect. Microbiol..

[10] Abbas A., Gruner M., Karohl J., Rose P.G., Joehlin-Price A., Stover D., Mahdi H. (2021). Case Report: Circulating Tumor DNA Fraction Analysis Using Ultra-Low-Pass Whole-Genome Sequencing Correlates Response to Chemoradiation and Recurrence in Stage IV Small-Cell Carcinoma of the Cervix—A Longitudinal Study. Front. Oncol..

[11] Chu T., Meng Y., Wu P., Li Z., Wen H., Ren F., Zou D., Lu H., Wu L., Zhou S. (2023). The prognosis of patients with small cell carcinoma of the cervix: A retrospective study of the SEER database and a Chinese multicentre registry. Lancet Oncol..

[12] Abeler V.M., Holm R., Nesland J.M., Kjørstad K.E. (1994). Small cell carcinoma of the cervix. A clinicopathologic study of 26 patients. Cancer.

[13] Satoh T., Takei Y., Treilleux I., Devouassoux-Shisheboran M., Ledermann J., Viswanathan A.N., Mahner S., Provencher D.M., Mileshkin L., Åvall-Lundqvist E. (2014). Gynecologic Cancer InterGroup (GCIG) consensus review for small cell carcinoma of the cervix. Int. J. Gynecol. Cancer.

[14] Lee S.W., Nam J.H., Kim D.Y., Kim J.H., Kim K.R., Kim Y.M., Kim Y.T. (2010). Unfavorable prognosis of small cell neuroendocrine carcinoma of the uterine cervix: A retrospective matched case-control study. Int. J. Gynecol. Cancer.

[15] Sevin B.U., Method M.W., Nadji M., Lu Y., Averette H.A. (1996). Efficacy of radical hysterectomy as treatment for patients with small cell carcinoma of the cervix. Cancer.

[16] Van Nagell J.R., Donaldson E.S., Wood E.G., Maruyama Y., Utley J. (1977). Small cell cancer of the uterine cervix. Cancer.

[17] Tan C.Y., Yang Q.L., Xu N., Wang H.J. (2024). Small cell neuroendocrine carcinoma of the cervix: An analysis for 5 cases and literature review. Asian J. Surg..

[18] Zhou C., Hayes M.M., Clement P.B., Thomson T.A. (1998). Small cell carcinoma of the uterine cervix: Cytologic findings in 13 cases. Cancer.

[19] Yang D.H., Kim J.K., Kim K.W., Bae S.J., Kim K.H., Cho K.S. (2004). MRI of small cell carcinoma of the uterine cervix with pathologic correlation. AJR Am. J. Roentgenol..

[20] Ambros R.A., Park J.S., Shah K.V., Kurman R.J. (1991). Evaluation of histologic, morphometric, and immunohistochemical criteria in the differential diagnosis of small cell carcinomas of the cervix with particular reference to human papillomavirus types 16 and 18. Mod. Pathol..

[21] Wistuba I.I., Thomas B., Behrens C., Onuki N., Lindberg G., Albores-Saavedra J., Gazdar A.F. (1999). Molecular abnormalities associated with endocrine tumors of the uterine cervix. Gynecol. Oncol..

[22] Albores-Saavedra J., Gersell D., Gilks C.B., Henson D.E., Lindberg G., Santiago H., Scully R.E., Silva E., Sobin L.H., Tavassoli F.J. (1997). Terminology of endocrine tumors of the uterine cervix: Results of a workshop sponsored by the College of American Pathologists and the National Cancer Institute. Arch. Pathol. Lab. Med..

[23] Paraghamian S.E., Longoria T.C., Eskander R.N. (2017). Metastatic small cell neuroendocrine carcinoma of the cervix treated with the PD-1 inhibitor, nivolumab: A case report. Gynecol. Oncol. Res. Pract..

[24] Abu-Rustum N.R., Yashar C.M., Arend R., Barber E., Bradley K., Brooks R., Campos S.M., Chino J., Chon H.S., Chu C. (2023). National Comprehensive Cancer Network (NCCN) Guidelines: Cervical Cancer, Version 1. https://www.nccn.org.

[25] Huang R., Yu L., Zheng C., Liang Q., Suye S., Yang X., Yin H., Ren Z., Shi L., Zhang Z. (2020). Diagnostic value of four neuroendocrine markers in small cell neuroendocrine carcinomas of the cervix: A meta-analysis. Sci. Rep..

[26] Kuji S., Endo A., Kubota M., Uekawa A., Kawakami F., Mikami Y., Koike J., Suzuki N. (2023). Immunosensitivity and specificity of insulinoma-associated protein 1 (INSM1) for neuroendocrine neoplasms of the uterine cervix. J. Gynecol. Oncol..

[27] Kim G., Kim M., Nam E.J., Lee J.Y., Park E. (2023). Application of Small Cell Lung Cancer Molecular Subtyping Markers to Small Cell Neuroendocrine Carcinoma of the Cervix: NEUROD1 as a Poor Prognostic Factor. Am. J. Surg. Pathol..

[28] Baedyananda F., Sasivimolrattana T., Chaiwongkot A., Varadarajan S., Bhattarakosol P. (2022). Role of HPV16 E1 in cervical carcinogenesis. Front. Cell Infect. Microbiol..

[29] (2007). Quadrivalent vaccine against human papillomavirus to prevent high-grade cervical lesions. N. Engl. J. Med..

[30] Pao C.C., Lin C.Y., Chang Y.L., Tseng C.J., Hsueh S. (1991). Human papillomaviruses and small cell carcinoma of the uterine cervix. Gynecol. Oncol..

[31] Hillman R.T., Cardnell R., Fujimoto J., Lee W.C., Zhang J., Byers L.A., Ramalingam P., Leitao M., Swisher E., Futreal P.A. (2020). Comparative genomics of high grade neuroendocrine carcinoma of the cervix. PLoS ONE.

[32] Kusakabe M., Taguchi A., Tanikawa M., Hoshi D., Tsuchimochi S., Qian X., Toyohara Y., Kawata A., Wagatsuma R., Yamaguchi K. (2023). Application of organoid culture from HPV18-positive small cell carcinoma of the uterine cervix for precision medicine. Cancer Med..

[33] Wang W., Zhang F., Li Y., Chen B., Gu Y., Shan Y., Li Y., Chen W., Jin Y., Pan L. (2023). Whole exome sequencing identifies common mutational landscape of cervix and endometrium small cell neuroendocrine carcinoma. Front. Oncol..

[34] Rusan M., Li Y.Y., Hammerman P.S. (2015). Genomic landscape of human papillomavirus-associated cancers. Clin. Cancer Res..

[35] Ojesina A.I., Lichtenstein L., Freeman S.S., Pedamallu C.S., Imaz-Rosshandler I., Pugh T.J., Cherniack A.D., Ambrogio L., Cibulskis K., Bertelsen B. (2014). Landscape of genomic alterations in cervical carcinomas. Nature.

[36] Wang H.L., Lu D.W. (2004). Detection of human papillomavirus DNA and expression of p16, Rb, and p53 proteins in small cell carcinomas of the uterine cervix. Am. J. Surg. Pathol..

[37] Masumoto N., Fujii T., Ishikawa M., Saito M., Iwata T., Fukuchi T., Susumu N., Mukai M., Kubushiro K., Tsukazaki K. (2003). P16 overexpression and human papillomavirus infection in small cell carcinoma of the uterine cervix. Hum. Pathol..

[38] Xing D., Zheng G., Schoolmeester J.K., Li Z., Pallavajjala A., Haley L., Conner M.G., Vang R., Hung C.F., Wu T.C. (2018). Next-generation Sequencing Reveals Recurrent Somatic Mutations in Small Cell Neuroendocrine Carcinoma of the Uterine Cervix. Am. J. Surg. Pathol..

[39] Stoler M.H., Mills S.E., Gersell D.J., Walker A.N. (1991). Small-cell neuroendocrine carcinoma of the cervix. A human papillomavirus type 18-associated cancer. Am. J. Surg. Pathol..

[40] Ordulu Z., Mino-Kenudson M., Young R.H., Van de Vijver K., Zannoni G.F., Félix A., Burandt E., Wong A., Nardi V., Oliva E. (2022). Morphologic and Molecular Heterogeneity of Cervical Neuroendocrine Neoplasia: A Report of 14 Cases. Am. J. Surg. Pathol..

[41] Albrecht L.V., Tejeda-Muñoz N., De Robertis E.M. (2021). Cell Biology of Canonical Wnt Signaling. Annu. Rev. Cell Dev. Biol..

[42] Eskander R.N., Elvin J., Gay L., Ross J.S., Miller V.A., Kurzrock R. (2020). Unique Genomic Landscape of High-Grade Neuroendocrine Cervical Carcinoma: Implications for Rethinking Current Treatment Paradigms. JCO Precis. Oncol..

[43] Ishida G.M., Kato N., Hayasaka T., Saito M., Kobayashi H., Katayama Y., Sasou S., Yaegashi N., Kurachi H., Motoyama T. (2004). Small cell neuroendocrine carcinomas of the uterine cervix: A histological, immunohistochemical, and molecular genetic study. Int. J. Gynecol. Pathol..

[44] Mannion C., Park W.S., Man Y.G., Zhuang Z., Albores-Saavedra J., Tavassoli F.A. (1998). Endocrine tumors of the cervix: Morphologic assessment, expression of human papillomavirus, and evaluation for loss of heterozygosity on 1p,3p, 11q, and 17p. Cancer.

[45] Frumovitz M., Burzawa J.K., Byers L.A., Lyons Y.A., Ramalingam P., Coleman R.L., Brown J. (2016). Sequencing of mutational hotspots in cancer-related genes in small cell neuroendocrine cervical cancer. Gynecol. Oncol..

[46] Cho S.Y., Choi M., Ban H.J., Lee C.H., Park S., Kim H., Kim Y.S., Lee Y.S., Lee J.Y. (2017). Cervical small cell neuroendocrine tumor mutation profiles via whole exome sequencing. Oncotarget.

[47] Lyons Y.A., Frumovitz M., Soliman P.T. (2014). Response to MEK inhibitor in small cell neuroendocrine carcinoma of the cervix with a KRAS mutation. Gynecol. Oncol. Rep..

[48] Huang L., Lin J.X., Yu Y.H., Zhang M.Y., Wang H.Y., Zheng M. (2012). Downregulation of six microRNAs is associated with advanced stage, lymph node metastasis and poor prognosis in small cell carcinoma of the cervix. PLoS ONE.

[49] Watkins T.B.K., Lim E.L., Petkovic M., Elizalde S., Birkbak N.J., Wilson G.A., Moore D.A., Grönroos E., Rowan A., Dewhurst S.M. (2020). Pervasive chromosomal instability and karyotype order in tumour evolution. Nature.

[50] Pei X., Xiang L., Chen W., Jiang W., Yin L., Shen X., Zhou X., Yang H. (2021). The next generation sequencing of cancer-related genes in small cell neuroendocrine carcinoma of the cervix. Gynecol. Oncol..

[51] Zhang S.W., Luo R.Z., Sun X.Y., Yang X., Yang H.X., Xiong S.P., Liu L.L. (2021). Co-expression of SOX2 and HR-HPV RISH predicts poor prognosis in small cell neuroendocrine carcinoma of the uterine cervix. BMC Cancer.

[52] Dubois F., Sidiropoulos N., Weischenfeldt J., Beroukhim R. (2022). Structural variations in cancer and the 3D genome. Nat. Rev. Cancer.

[53] Leardini D., Messelodi D., Muratore E., Baccelli F., Bertuccio S.N., Anselmi L., Pession A., Masetti R. (2022). Role of CBL Mutations in Cancer and Non-Malignant Phenotype. Cancers.

[54] Xiao Y., Chen Y., Peng A., Dong J. (2022). The phosphatase CTDSPL2 is phosphorylated in mitosis and a target for restraining tumor growth and motility in pancreatic cancer. Cancer Lett..

[55] Yang B., Wu A., Hu Y., Tao C., Wang J.M., Lu Y., Xing R. (2019). Mucin 17 inhibits the progression of human gastric cancer by limiting inflammatory responses through a MYH9-p53-RhoA regulatory feedback loop. J. Exp. Clin. Cancer Res..

[56] Berindan-Neagoe I., Monroig Pdel C., Pasculli B., Calin G.A. (2014). MicroRNAome genome: A treasure for cancer diagnosis and therapy. CA Cancer J. Clin..

[57] Chen T.C., Huang H.J., Wang T.Y., Yang L.Y., Chen C.H., Cheng Y.M., Liou W.H., Hsu S.T., Wen K.C., Ou Y.C. (2015). Primary surgery versus primary radiation therapy for FIGO stages I-II small cell carcinoma of the uterine cervix: A retrospective Taiwanese Gynecologic Oncology Group study. Gynecol. Oncol..

[58] Tangjitgamol S., Ramirez P.T., Sun C.C., See H.T., Jhingran A., Kavanagh J.J., Deavers M.T. (2005). Expression of HER-2/neu, epidermal growth factor receptor, vascular endothelial growth factor, cyclooxygenase-2, estrogen receptor, and progesterone receptor in small cell and large cell neuroendocrine carcinoma of the uterine cervix: A clinicopathologic and prognostic study. Int. J. Gynecol. Cancer.

[59] Inzani F., Santoro A., Angelico G., Feraco A., Spadola S., Arciuolo D., Valente M., Carlino A., Piermattei A., Scaglione G. (2020). Neuroendocrine Carcinoma of the Uterine Cervix: A Clinicopathologic and Immunohistochemical Study with Focus on Novel Markers (Sst2-Sst5). Cancers.

[60] Qiu H., Su N., Wang J., Yan S., Li J. (2023). Quantitative proteomics analysis in small cell carcinoma of cervix reveals novel therapeutic targets. Clin. Proteomics.

[61] Sherr C.J. (1996). Cancer cell cycles. Science.

[62] Herrington C.S., Graham D., Southern S.A., Bramdev A., Chetty R. (1999). Loss of retinoblastoma protein expression is frequent in small cell neuroendocrine carcinoma of the cervix and is unrelated to HPV type. Hum. Pathol..

[63] Sherr C.J., McCormick F. (2002). The RB and p53 pathways in cancer. Cancer Cell.

[64] Straughn J.M., Richter H.E., Conner M.G., Meleth S., Barnes M.N. (2001). Predictors of outcome in small cell carcinoma of the cervix--a case series. Gynecol. Oncol..

[65] Zarka T.A., Han A.C., Edelson M.I., Rosenblum N.G. (2003). Expression of cadherins, p53, and BCL2 in small cell carcinomas of the cervix: Potential tumor suppressor role for N-cadherin. Int. J. Gynecol. Cancer.

[66] Yamaguchi H., Hsu J.M., Yang W.H., Hung M.C. (2022). Mechanisms regulating PD-L1 expression in cancers and associated opportunities for novel small-molecule therapeutics. Nat. Rev. Clin. Oncol..

[67] Chen L., Yang F., Feng T., Wu S., Li K., Pang J., Shi X., Liang Z. (2021). PD-L1, Mismatch Repair Protein, and NTRK Immunohistochemical Expression in Cervical Small Cell Neuroendocrine Carcinoma. Front. Oncol..

[68] Morgan S., Slodkowska E., Parra-Herran C., Mirkovic J. (2019). PD-L1, RB1 and mismatch repair protein immunohistochemical expression in neuroendocrine carcinoma, small cell type, of the uterine cervix. Histopathology.

[69] Sun X., Liu L., Wan T., Huang Q., Chen J., Luo R., Liu J. (2022). The prognostic impact of the immune microenvironment in small-cell neuroendocrine carcinoma of the uterine cervix: PD-L1 and immune cell subtypes. Cancer Cell Int..

[70] Zandarashvili L., Langelier M.F., Velagapudi U.K., Hancock M.A., Steffen J.D., Billur R., Hannan Z.M., Wicks A.J., Krastev D.B., Pettitt S.J. (2020). Structural basis for allosteric PARP-1 retention on DNA breaks. Science.

[71] Carroll M.R., Ramalingam P., Salvo G., Fujimoto J., Solis Soto L.M., Phoolcharoen N., Hillman R.T., Cardnell R., Byers L., Frumovitz M. (2020). Evaluation of PARP and PDL-1 as potential therapeutic targets for women with high-grade neuroendocrine carcinomas of the cervix. Int. J. Gynecol. Cancer.

[72] Quinn A.M., Blackhall F., Wilson G., Danson S., Clamp A., Ashcroft L., Brierley J., Hasleton P. (2012). Extrapulmonary small cell carcinoma: A clinicopathological study with identification of potential diagnostic mimics. Histopathology.

[73] Margolis B., Tergas A.I., Chen L., Hou J.Y., Burke W.M., Hu J.C., Ananth C.V., Neugut A.I., Hershman D.L., Wright J.D. (2016). Natural history and outcome of neuroendocrine carcinoma of the cervix. Gynecol. Oncol..

[74] Sykes A.J., Shanks J.H., Davidson S.E. (1999). Small cell carcinoma of the uterine cervix: A clinicopathological review. Int. J. Oncol..

[75] Cohen J.G., Kapp D.S., Shin J.Y., Urban R., Sherman A.E., Chen L.M., Osann K., Chan J.K. (2010). Small cell carcinoma of the cervix: Treatment and survival outcomes of 188 patients. Am. J. Obstet. Gynecol..

[76] Kawamura M., Koide Y., Murai T., Ishihara S., Takase Y., Murao T., Okazaki D., Yamaguchi T., Uchiyama K., Itoh Y. (2021). The importance of choosing the right strategy to treat small cell carcinoma of the cervix: A comparative analysis of treatments. BMC Cancer.

[77] Kuji S., Hirashima Y., Nakayama H., Nishio S., Otsuki T., Nagamitsu Y., Tanaka N., Ito K., Teramoto N., Yamada T. (2013). Diagnosis, clinicopathologic features, treatment, and prognosis of small cell carcinoma of the uterine cervix; Kansai Clinical Oncology Group/Intergroup study in Japan. Gynecol. Oncol..

[78] Huang L., Liao L.M., Liu A.W., Wu J.B., Cheng X.L., Lin J.X., Zheng M. (2014). Analysis of the impact of platinum-based combination chemotherapy in small cell cervical carcinoma: A multicenter retrospective study in Chinese patients. BMC Cancer.

[79] Li X., Yang R., Jia Y., Zhou J., Ma D., Li S. (2015). Prognostic risk factors for small cell carcinoma of the cervix and impact of platinum-based neoadjuvant chemotherapy. Int. J. Gynaecol. Obstet..

[80] Li J., Ouyang Y., Tao Y., Wang L., Li M., Gao L., Cao X. (2020). Small cell carcinoma of the uterine cervix: A multi-institutional experience. Int. J. Gynecol. Cancer.

[81] Wang K.L., Chang T.C., Jung S.M., Chen C.H., Cheng Y.M., Wu H.H., Liou W.S., Hsu S.T., Ou Y.C., Yeh L.S. (2012). Primary treatment and prognostic factors of small cell neuroendocrine carcinoma of the uterine cervix: A Taiwanese Gynecologic Oncology Group study. Eur. J. Cancer.

[82] Song T., Wan Q., Fang M., Zhan W., Xu H., Shou H. (2020). Trends and predictors of survival for small cell carcinoma of the cervix uteri: A SEER population study. Eur. J. Obstet. Gynecol. Reprod. Biol..

[83] Zhang Q., Xiong Y., Ye J., Zhang L., Li L. (2018). Influence of clinicopathological characteristics and comprehensive treatment models on the prognosis of small cell carcinoma of the cervix: A systematic review and meta-analysis. PLoS ONE.

[84] Liao L.M., Zhang X., Ren Y.F., Sun X.Y., Di N., Zhou N., Pan R.K., Ma S.H., Zhou L.X. (2012). Chromogranin A (CgA) as poor prognostic factor in patients with small cell carcinoma of the cervix: Results of a retrospective study of 293 patients. PLoS ONE.

[85] Chang T.C., Lai C.H., Tseng C.J., Hsueh S., Huang K.G., Chou H.H. (1998). Prognostic factors in surgically treated small cell cervical carcinoma followed by adjuvant chemotherapy. Cancer.

[86] Hoskins P.J., Swenerton K.D., Pike J.A., Lim P., Aquino-Parsons C., Wong F., Lee N. (2003). Small-cell carcinoma of the cervix: Fourteen years of experience at a single institution using a combined-modality regimen of involved-field irradiation and platinum-based combination chemotherapy. J. Clin. Oncol..

[87] Lee J.M., Lee K.B., Nam J.H., Ryu S.Y., Bae D.S., Park J.T., Kim S.C., Cha S.D., Kim K.R., Song S.Y. (2008). Prognostic factors in FIGO stage IB-IIA small cell neuroendocrine carcinoma of the uterine cervix treated surgically: Results of a multi-center retrospective Korean study. Ann. Oncol..

[88] Viswanathan A.N., Deavers M.T., Jhingran A., Ramirez P.T., Levenback C., Eifel P.J. (2004). Small cell neuroendocrine carcinoma of the cervix: Outcome and patterns of recurrence. Gynecol. Oncol..

[89] Chan J.K., Loizzi V., Burger R.A., Rutgers J., Monk B.J. (2003). Prognostic factors in neuroendocrine small cell cervical carcinoma: A multivariate analysis. Cancer.

[90] Huang C.Y., Chen Y.L., Chu T.C., Cheng W.F., Hsieh C.Y., Lin M.C. (2009). Prognostic factors in women with early stage small cell carcinoma of the uterine cervix. Oncol. Res..

[91] Wang P.H., Liu Y.C., Lai C.R., Chao H.T., Yuan C.C., Yu K.J. (1998). Small cell carcinoma of the cervix: Analysis of clinical and pathological findings. Eur. J. Gynaecol. Oncol..

[92] Shen T., Jiang Y.H., Zou Y.Y., Qiu F.F., Qiu X.S., You K.Y. (2019). Postoperative adjuvant radiation improves local control in surgically treated FIGO stage I-II small cell carcinoma of the cervix. Radiat. Oncol..

[93] Wang K.L., Yang Y.C., Wang T.Y., Chen J.R., Chen T.C., Chen H.S., Su T.H., Wang K.G. (2006). Neuroendocrine carcinoma of the uterine cervix: A clinicopathologic retrospective study of 31 cases with prognostic implications. J. Chemother..

[94] Chen Y., Chen J., Lin X., Zheng J., Li S., Zheng X. (2022). A Prognostic Nomogram for Predicting Overall Survival in Patients With Small-Cell Carcinoma of the Uterine Cervix: A SEER Population-Based Study. Technol. Cancer Res. Treat..

[95] Intaraphet S., Kasatpibal N., Siriaunkgul S., Chandacham A., Sukpan K., Patumanond J. (2014). Prognostic factors for small cell neuroendocrine carcinoma of the uterine cervix: An institutional experience. Int. J. Gynecol. Cancer.

[96] Gadducci A., Carinelli S., Aletti G. (2017). Neuroendrocrine tumors of the uterine cervix: A therapeutic challenge for gynecologic oncologists. Gynecol. Oncol..

[97] Gennigens C., De Cuypere M., Hermesse J., Kridelka F., Jerusalem G. (2021). Optimal treatment in locally advanced cervical cancer. Expert. Rev. Anticancer. Ther..

[98] Uwins C., Patel H., Prakash Bhandoria G., Butler-Manuel S., Tailor A., Ellis P., Chatterjee J. (2021). Laparoscopic and Robotic Surgery for Endometrial and Cervical Cancer. Clin. Oncol. R. Coll. Radiol..

[99] O’Hanlan K.A., Goldberg G.L., Jones J.G., Runowicz C.D., Ehrlich L., Rodriguez-Rodriguez L. (1991). Adjuvant therapy for neuroendocrine small cell carcinoma of the cervix: Review of the literature. Gynecol. Oncol..

[100] Hoskins P.J., Wong F., Swenerton K.D., Pike J.A., Manji M., McMurtrie E., Acker B., Le Riche J. (1995). Small cell carcinoma of the cervix treated with concurrent radiotherapy, cisplatin, and etoposide. Gynecol. Oncol..

[101] Pei X., Xiang L., Ye S., He T., Cheng Y., Yang W., Wu X., Yang H. (2017). Cycles of cisplatin and etoposide affect treatment outcomes in patients with FIGO stage I-II small cell neuroendocrine carcinoma of the cervix. Gynecol. Oncol..

[102] Allen J.W., Moon J., Redman M., Gadgeel S.M., Kelly K., Mack P.C., Saba H.M., Mohamed M.K., Jahanzeb M., Gandara D.R. (2014). Southwest Oncology Group S0802: A randomized, phase II trial of weekly topotecan with and without ziv-aflibercept in patients with platinum-treated small-cell lung cancer. J. Clin. Oncol..

[103] Qiu H., Su N., Yan S., Li J. (2023). Real-world Efficacy Data on Anti-Angiogenic Drugs in Recurrent Small Cell Cervical Carcinoma: A Retrospective Study. Technol. Cancer Res. Treat..

[104] Frumovitz M., Chisholm G.B., Jhingran A., Ramalingam P., Flores-Legarreta A., Bhosale P., Gonzales N.R., Hillman R.T., Salvo G. (2023). Combination therapy with topotecan, paclitaxel, and bevacizumab improves progression-free survival in patients with recurrent high-grade neuroendocrine cervical cancer: A Neuroendocrine Cervical Tumor Registry (NeCTuR) study. Am. J. Obstet. Gynecol..

[105] Frumovitz M., Munsell M.F., Burzawa J.K., Byers L.A., Ramalingam P., Brown J., Coleman R.L. (2017). Combination therapy with topotecan, paclitaxel, and bevacizumab improves progression-free survival in recurrent small cell neuroendocrine carcinoma of the cervix. Gynecol. Oncol..

[106] Lopez-Chavez A., Thomas A., Rajan A., Raffeld M., Morrow B., Kelly R., Carter C.A., Guha U., Killian K., Lau C.C. (2015). Molecular profiling and targeted therapy for advanced thoracic malignancies: A biomarker-derived, multiarm, multihistology phase II basket trial. J. Clin. Oncol..

[107] Pietanza M.C., Waqar S.N., Krug L.M., Dowlati A., Hann C.L., Chiappori A., Owonikoko T.K., Woo K.M., Cardnell R.J., Fujimoto J. (2018). Randomized, Double-Blind, Phase II Study of Temozolomide in Combination With Either Veliparib or Placebo in Patients With Relapsed-Sensitive or Refractory Small-Cell Lung Cancer. J. Clin. Oncol..

[108] de Bono J., Ramanathan R.K., Mina L., Chugh R., Glaspy J., Rafii S., Kaye S., Sachdev J., Heymach J., Smith D.C. (2017). Phase I, Dose-Escalation, Two-Part Trial of the PARP Inhibitor Talazoparib in Patients with Advanced Germline BRCA1/2 Mutations and Selected Sporadic Cancers. Cancer Discov..

[109] Thomas A., Redon C.E., Sciuto L., Padiernos E., Ji J., Lee M.J., Yuno A., Lee S., Zhang Y., Tran L. (2018). Phase I Study of ATR Inhibitor M6620 in Combination With Topotecan in Patients With Advanced Solid Tumors. J. Clin. Oncol..

[110] Jones R., Plummer R., Moreno V., Carter L., Roda D., Garralda E., Kristeleit R., Sarker D., Arkenau T., Roxburgh P. (2023). A Phase I/II Trial of Oral SRA737 (a Chk1 Inhibitor) Given in Combination with Low-Dose Gemcitabine in Patients with Advanced Cancer. Clin. Cancer Res..

[111] Bauer T.M., Moore K.N., Rader J.S., Simpkins F., Mita A.C., Beck J.T., Hart L., Chu Q., Oza A., Tinker A.V. (2023). A Phase Ib Study Assessing the Safety, Tolerability, and Efficacy of the First-in-Class Wee1 Inhibitor Adavosertib (AZD1775) as Monotherapy in Patients with Advanced Solid Tumors. Target. Oncol..

[112] Beltran H., Oromendia C., Danila D.C., Montgomery B., Hoimes C., Szmulewitz R.Z., Vaishampayan U., Armstrong A.J., Stein M., Pinski J. (2019). A Phase II Trial of the Aurora Kinase A Inhibitor Alisertib for Patients with Castration-resistant and Neuroendocrine Prostate Cancer: Efficacy and Biomarkers. Clin. Cancer Res..

[113] Chiappori A.A., Williams C.C., Gray J.E., Tanvetyanon T., Haura E.B., Creelan B.C., Thapa R., Chen D.T., Simon G.R., Bepler G. (2019). Randomized-controlled phase II trial of salvage chemotherapy after immunization with a TP53-transfected dendritic cell-based vaccine (Ad.p53-DC) in patients with recurrent small cell lung cancer. Cancer Immunol. Immunother..

[114] Bauer T.M., Besse B., Martinez-Marti A., Trigo J.M., Moreno V., Garrido P., Ferron-Brady G., Wu Y., Park J., Collingwood T. (2019). Phase I, Open-Label, Dose-Escalation Study of the Safety, Pharmacokinetics, Pharmacodynamics, and Efficacy of GSK2879552 in Relapsed/Refractory SCLC. J. Thorac. Oncol..

[115] Rudin C.M., Awad M.M., Navarro A., Gottfried M., Peters S., Csőszi T., Cheema P.K., Rodriguez-Abreu D., Wollner M., Yang J.C. (2020). Pembrolizumab or Placebo Plus Etoposide and Platinum as First-Line Therapy for Extensive-Stage Small-Cell Lung Cancer: Randomized, Double-Blind, Phase III KEYNOTE-604 Study. J. Clin. Oncol..

[116] Luke J.J., Patel M.R., Blumenschein G.R., Hamilton E., Chmielowski B., Ulahannan S.V., Connolly R.M., Santa-Maria C.A., Wang J., Bahadur S.W. (2023). The PD-1- and LAG-3-targeting bispecific molecule tebotelimab in solid tumors and hematologic cancers: A phase 1 trial. Nat. Med..

[117] Marchocki Z., Swift B., Covens A. (2022). Small Cell and Other Rare Histologic Types of Cervical Cancer. Curr. Oncol. Rep..

[118] Ngoi N.Y., Sundararajan V., Tan D.S. (2020). Exploiting replicative stress in gynecological cancers as a therapeutic strategy. Int. J. Gynecol. Cancer.

[119] Banerjee N.S., Moore D., Parker C.J., Broker T.R., Chow L.T. (2019). Targeting DNA Damage Response as a Strategy to Treat HPV Infections. Int. J. Mol. Sci..

[120] Tung H.J., Wang C.C., Liu F.Y., Lai C.H. (2021). Complete remission of advanced and recurrent cervical cancer with pembrolizumab treatment- 3 case reports. Taiwan. J. Obstet. Gynecol..

[121] Paterniti T.A., Dorr K., Ullah A., White J., Williams H., Ghamande S. (2021). Complete Response to Combination Nivolumab and Ipilimumab in Recurrent Neuroendocrine Carcinoma of the Cervix. Obstet. Gynecol..

[122] Frumovitz M., Westin S.N., Salvo G., Zarifa A., Xu M., Yap T.A., Rodon A.J., Karp D.D., Abonofal A., Jazaeri A.A. (2020). Phase II study of pembrolizumab efficacy and safety in women with recurrent small cell neuroendocrine carcinoma of the lower genital tract. Gynecol. Oncol..

[123] Sato H., Niimi A., Yasuhara T., Permata T.B.M., Hagiwara Y., Isono M., Nuryadi E., Sekine R., Oike T., Kakoti S. (2017). DNA double-strand break repair pathway regulates PD-L1 expression in cancer cells. Nat. Commun..

[124] Naidoo J., Teo M.Y., Deady S., Comber H., Calvert P. (2013). Should patients with extrapulmonary small-cell carcinoma receive prophylactic cranial irradiation?. J. Thorac. Oncol..

[125] Weed J.C., Graff A.T., Shoup B., Tawfik O. (2003). Small cell undifferentiated (neuroendocrine) carcinoma of the uterine cervix. J. Am. Coll. Surg..

[126] Rehman A.U., Khan P., Maurya S.K., Siddiqui J.A., Santamaria-Barria J.A., Batra S.K., Nasser M.W. (2022). Liquid biopsies to occult brain metastasis. Mol. Cancer.

[127] De Mattos-Arruda L., Mayor R., Ng C.K.Y., Weigelt B., Martínez-Ricarte F., Torrejon D., Oliveira M., Arias A., Raventos C., Tang J. (2015). Cerebrospinal fluid-derived circulating tumour DNA better represents the genomic alterations of brain tumours than plasma. Nat. Commun..

[128] Tanaka M., Kondo J., Kaneko K., Endo H., Onuma K., Coppo R., Masuda M., Kamiura S., Yoshino K., Ueda Y. (2021). Heterogenous chemosensitivity of a panel of organoid lines derived from small cell neuroendocrine carcinoma of the uterine cervix. Hum. Cell.

[129] Nakajima A., Endo H., Okuyama H., Kiyohara Y., Kimura T., Kamiura S., Hiraoka M., Inoue M. (2015). Radiation sensitivity assay with a panel of patient-derived spheroids of small cell carcinoma of the cervix. Int. J. Cancer.

[130] Tang F., Xu D., Wang S., Wong C.K., Martinez-Fundichely A., Lee C.J., Cohen S., Park J., Hill C.E., Eng K. (2022). Chromatin profiles classify castration-resistant prostate cancers suggesting therapeutic targets. Science.

[131] Gay C.M., Stewart C.A., Park E.M., Diao L., Groves S.M., Heeke S., Nabet B.Y., Fujimoto J., Solis L.M., Lu W. (2021). Patterns of transcription factor programs and immune pathway activation define four major subtypes of SCLC with distinct therapeutic vulnerabilities. Cancer Cell.

